# Successful Plasma Exchange for Acute Pancreatitis Complicated With Hypertriglyceridemia: A Case Report

**DOI:** 10.1177/2324709615605635

**Published:** 2015-09-10

**Authors:** Shuji Takahira, Hiromichi Suzuki, Yusuke Watanabe, Hunsook Kin, Yoshitaka Ooya, Yasumasa Sekine, Kenichiro Sonoda, Hiroshi Ogawa, Yushi Nomura, Hiroshi Takane, Youhei Tsuchiya, Isao Tsukamoto, Manabu Nemoto

**Affiliations:** 1 Saitama Medical University International Medical Center, Saitama, Japan

**Keywords:** acute pancreatitis, hypertriglyceridemia, plasma exchange, membrane clotting, warming

## Abstract

A 33-year-old male with acute pancreatitis induced by hypertriglyceridemia had problems during treatment with plasma exchange. The hypercoagulable state was prevented by introducing innovative methods for cleaning and warming of the circuit and dialyzer. This enabled successful therapy, and the patient fully recovered from life-threatening acute pancreatitis.

## Introduction

Although severe hypertriglyceridemia is found only in 1% to 7% of all cases of acute pancreatitis, it remains as one of the challenging problems in critical care medicine. Among the modalities for treatment, plasma exchange (PE)^1,2^ has been considered promising.

In this article, we report a case of a patient who experienced an increase in blood viscosity and membrane clotting during blood purification therapy for hypertriglyceridemia complicated with acute pancreatitis. Also, successful treatment that was achieved with innovative methods for prevention of hypercoagulability in a circuit during PE is described.

## Case Report

A 33-year-old male was admitted to the emergency department of our hospital for severe abdominal pain radiating to the back, which had started on the previous day. He had visited another hospital and received analgesics without relief. He had a past history of surgery for removal of a nonfunctioning pituitary tumor 14 years earlier, and he was followed at another hospital. Also, he had an appendectomy 10 years earlier and had been taking 10 mg hydrocortisol orally and receiving 2 sprays of desmopressin acetate daily. There was a family history of hyperlipidemia, and his parents are on antilipid medications.

On physical examination, he was alert; body temperature was 38.1°C, arterial blood pressure was 145/101 mm Hg, and heart rate was 132/min. There was neither jaundice nor anemia. The abdomen was flat and found to have rebound tenderness on the epigastrium. Laboratory findings are shown in Table 1. Serum triglyceride was over 10 000 mg/dL, and total cholesterol was 910 mg/dL. Serum corrected calcium was7.1 mg/dL. Electrocardiography and chest X-ray were normal.

**Table 1.** Laboratory Findings.

Peripheral blood	Serum chemistry
WBC: 28.50 ×1000/µ	TP: 5.0 g/dL
RBC: 5.25 × 10^4^/µ	ALB: 3.5 g/dL
Hb: 21.5 g/dL	CK: 35 U/L
HCT: 45.5%	AST: 25 U/L
PLT: 47.3 × 10^4^/µ	ALT: 55 U/L
NEUT: 88.7%	LDH: 280 U/L
Coagulation test	AMY: 420 U/L
APTT: 35.0 seconds	Cr: 0.37 mg/dL
PT: 11.8 seconds	UA: 3.7 mg/dL
PT%: 99%	BUN: 8 mg/dL
PT-INR: 1.01	Na: 117 mEq/L
D-dimer: 0.50 µg/mL	Cl: 88 mEq/L
Blood gas analysis	K: 3.5 mEq/L
pH: 7.472	Ca (corrected): 7.1 mg/dL
pO_2_: 67.0 mm Hg	T-Bil: 0.3 mg/dL
pCO_2_: 31.7 mm Hg	CRP: 8.327 mg/dL
HCO_3_^−^: 22.7 mm Hg	TG: 10 000 mg/dL ↑
	T-cho: 910 mg/dL
	PG: 280 mg/dL
	HbA1c (JDS): 7.3％
	HBS Ag: Negative
	HCV Ab: Negative

Abbreviations: WBC, white blood cell; RBC, red blood cell; Hb, hemoglobin; HCT, hematocrit; PLT, platelet; NEUT, neutrophils; APTT, activated partial thromboplastin time; PT, prothrombin time; INR, international normalized ratio; TP, total protein; ALB, albumin; CK, creating kinase; AST, aspartate aminotransferase; ALT, alanine aminotransferase; LDH, lactate dehydrogenase; AMY, amylase; Cr, creatinine; UA, uric acid; BUN, blood urea nitrogen; T-Bil, total bilirubin; CRP, C-reactive protein; TG, total glyceride; T-cho, total cholesterol; HBS Ag, *hepatitis* B surface antigen; HCV Ab, hepatitis C virus antibodies; PG, plasma glucose.

**Figure 1.**
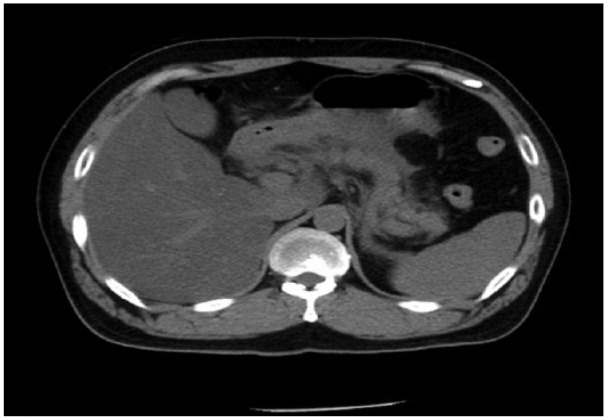
Computed tomography scan showing highly enlarged pancreatic parenchyma with peripheral fluid accumulation, bilateral pleural effusion, and right lower lobe atelectasis. Findings were consistent with acute pancreatitis.

Figure 1 shows a computed tomography scan of this patient at admission. The findings included a highly enlarged pancreatic parenchyma with peripheral fluid accumulation, bilateral pleural effusion, and right lower lobe atelectasis, which were consistent with acute pancreatitis. The patient was diagnosed with acute pancreatitis; Ranson’s score was 2, APACHE (acute physiology and chronic health evaluation) II score was 8, and Glasgow score was 3 at 48 hours.

Because hypertriglyceridemia was considered to be the cause of acute pancreatitis, a simple PE was performed using fresh frozen plasma (FFP). This was considered to be an appropriate purification method, as well as for removing lipids including triglycerides and humoral mediators.

The patient was admitted to the intensive care unit and oral food intake was prohibited. A protease inhibitor (FOY; Torii Co, Tokyo, Japan) was administered for acute pancreatitis in combination with antibiotics including carbapenem^3^ for severe pancreatitis, and buprenorphine was continuously administered to control abdominal pain associated with pancreatitis. Rapid-acting insulin was continuously administered to control high blood glucose. The purification method employed was a simple PE using a PE-08 (Toray Co, Tokyo, Japan) instrument with low-molecular-weight heparin, 800 units/hour, as anticoagulation therapy.

A central venous catheter was used and fluid balance was monitored. Forty units of FFP were used as the replacement fluid, and 2300 mL of plasma was exchanged. We used FFP instead of 5% albumin solution because we were concerned about the loss of immunoglobulin and coagulation factors. The duration of the PE was 240 minutes. PE was performed only twice, after which continuous hemofiltration as a nonrenal indication was performed to treat acute pancreatitis.^4^ From day 4 of hospitalization, continuous tube feeding with enteral nutrition support and intravenous hyperalimentation were started.^5^ Administration of hydrocortisone sodium succinate at 100 mg/day was started in order to prevent dyspituitarism. In light of improvement of the patient’s general condition, rehabilitation was started on day 6 of hospitalization.

**Figure 2.**
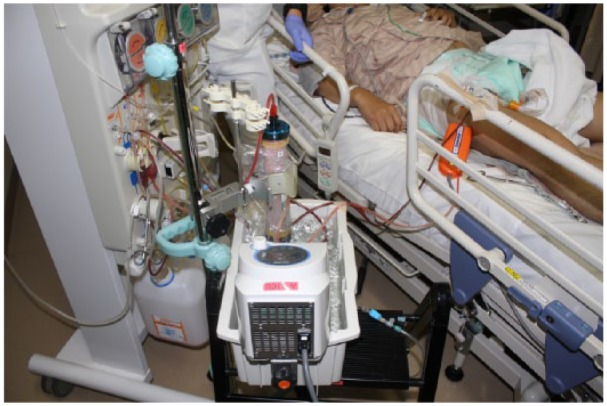
The circuit and dialyzer warmed up with vinyl cover and hot water.

After PE was started, clotting was found in the dialyzer. Cleaning with saline solution was conducted to prevent further clotting. After cleaning, the negative pressure gradient across the membrane was restored, thereby allowing successful PE. Besides, to prevent clotting due to lipid coagulation (Figure 2), the dialyzer was additionally covered with vinyl and heated with warm water (42°C). This hypercoagulability of the patient seemed to be enigmatic. After warming the circuit and connecting the dialyzer, clotting of blood disappeared indicating the presence of cold agglutinin. However, repeated examinations of blood for cold agglutinin were negative. On day 8 of hospitalization, the patient started to void urine spontaneously and was weaned from continuous hemodiafiltration. On day 27 of hospitalization, the patient was transferred to another hospital for detailed examination of his hyperlipidemia.

With these innovative methods, the patient achieved full recovery from life-threatening acute pancreatitis.

## Discussion

We report here a case of successful PE with circuit cleaning and heating for resolution of blood coagulation due to acute pancreatitis induced by hypertriglyceridemia. PE for reduction of serum triglyceridemia has been acknowledged as providing benefits to patients with hypertriglyceridemia.^6^ Especially in cases complicated by acute pancreatitis, PE has been recommended for use in therapeutic apheresis.^7^ During PE in our patient, unexpected accidents were encountered related to coagulation of the circuit and dialyzer.

### Cleaning With Saline Solution During Plasma Exchange

Many factors such as histamine, bradykinin, tumor necrosis factor-α, interleukin-1, fibrinogen degradation product, thrombin, and platelet activating factor are involved in hypercoagulability in acute pancreatitis due to hypertriglyceridemia.^8^ A simple PE was used to treat the patient, and blood cell and serum components were separated using a membrane plasma separator. Removing polarized molecules such as fatty acids and stabilizing lipids in plasma requires binding to proteins, which can result in large molecules. Indeed, the chylomicron particle system, which represents a major component of a triglyceride transporter lipoprotein, averages 80 nm in size. Given that the maximum pore diameter of the plasma separation membrane is only 30 nm, many chylomicron particles are often too large to pass, representing impermeable substances in the plasma separation process.

A concentrated polarization layer of impermeable substances can form on the surface of hollow fiber membranes. When the concentration of chylomicrons increases to a value greater than the solubility limit, a gel polarization layer is formed. Formation of such a layer is believed to not only reduce filtration pressure but also increase inlet pressure of the plasma separator through narrowing of the hollow fiber.^9^

### Heating During Plasma Exchange

As an additional preventative measure against clotting, the dialyzer was heated during PE therapy. The dialyzer was covered with vinyl and heated with warm water at 42°C, a temperature selected in consideration of the risk of irreversible changes in cells due to protein denaturation. A previous report cited safe performance of blood purification through heating the blood in the circuit and replacement fluid. In that study, the inside of the circuit was heated using plaster bandages and aluminum foil to prevent intracircuit coagulation during PE and ensure continuous hemodiafiltration in patients with cold agglutinin disease.^10^

Following the cleaning with saline and warming of the circuit, PE was continued without interruption.

In patients with severe hypertriglyceridemia, the hypercoagulable state and impaired fibrinolysis have been reported by some investigators with mixed results.^11,12^ In our investigations, no definite factors responsible for the hypercoagulable state were found.

## Conclusion

We experienced a case in which a patient’s life was saved by performing circuit cleaning and heating during PE for acute pancreatitis associated with hypertriglyceridemia. Circuit cleaning and heating at 42°C was demonstrated to be an effective method for relieving dialyzer clogging during total PE.
